# Toward microfluidic SERS and EC-SERS applications via tunable gold films over nanospheres

**DOI:** 10.1186/s11671-023-03851-3

**Published:** 2023-05-03

**Authors:** Alexandra Falamas, Denisa Cuibus, Nicoleta Tosa, Ioana Brezestean, Cristina M. Muntean, Karolina Milenko, Elizaveta Vereshchagina, Rebeca Moldovan, Ede Bodoki, Cosmin Farcau

**Affiliations:** 1 National Institute for Research and Development of Isotopic and Molecular Technologies, 67-103 Donat, 400293 Cluj-Napoca, Romania; 2 Department of Smart Sensors and Microsystems, SINTEF Digital, Gaustadalléen 23C, 0373 Oslo, Norway; 3 Analytical Chemistry Department, Faculty of Pharmacy, Iuliu Hațieganu” University of Medicine and Pharmacy, 4 Louis Pasteur, 400349 Cluj-Napoca, Romania; 4 Institute for Interdisciplinary Research in Nano-Bio-Sciences, Babes-Bolyai University, 42 T Laurian, 400271 Cluj-Napoca, Romania

**Keywords:** Gold films over nanospheres (AuFoN), Surface-enhanced Raman scattering (SERS), Electrochemical (EC)-SERS, Microfluidic SERS, Finite-difference time-domain (FDTD) simulations

## Abstract

Many promising applications of surface-enhanced Raman scattering (SERS), such as microfluidic SERS and electrochemical (EC)-SERS, require immersion of plasmonic nanostructured films in aqueous media. Correlational investigations of the optical response and SERS efficiency of solid SERS substrates immersed in water are absent in the literature. This work presents an approach for tuning the efficiency of gold films over nanospheres (AuFoN) as SERS substrates for applications in aqueous environment. AuFoN are fabricated by convective self-assembly of colloidal polystyrene nanospheres of various diameters (300–800 nm), followed by magnetron sputtering of gold films. The optical reflectance of the AuFoN and Finite-Difference Time-Domain simulations in both water and air reveal the dependence of the surface plasmon band on nanospheres’ diameter and environment. SERS enhancement of a common Raman reporter on AuFoN immersed in water is analyzed under 785 nm laser excitation, but also using the 633 nm line for the films in air. The provided correlations between the SERS efficiency and optical response in both air and water indicate the best structural parameters for high SERS efficiency and highlight a route for predicting and optimizing the SERS response of AuFoN in water based on the behavior in air, which is more practical. Finally, the AuFoN are successfully tested as electrodes for EC-SERS detection of the thiabendazole pesticide and as SERS substrates integrated in a flow-through microchannel format. The obtained results represent an important step toward the development of microfluidic EC-SERS devices for sensing applications.

**Supplementary Information:**

The online version contains supplementary material available at 10.1186/s11671-023-03851-3.

## Introduction

Surface-enhanced Raman scattering (SERS) has attracted a significant amount of theoretical and experimental research aimed at expanding the capabilities of the technique, such as molecular detection and identification, as well as substrate design and fabrication [1]. The enhancement of the Raman signal, inherently weak, but rich in structural information, is obtained mainly due the excitation of localized surface plasmon resonances (LSPRs) on nanostructured surfaces, which allow the concentration of light preferentially in the gaps and sharp features of such plasmonic nanomaterials [2, 3]. A molecule that is in the vicinity of a nanostructured metallic surface undergoes a local field enhancement due to the coupling of the incident excitation light with the surface plasmon oscillations of the metallic nanostructures, as well as a re-radiation enhancement due to the coupling between the Raman scattered photons and the surface plasmons. Owing to the high enhancement factors obtained in SERS, the unique vibrational fingerprint signal associated with chemical and structural information, the sensitivity, selectivity, and portability of the technique, SERS has been proposed for the detection of numerous target compounds including cancer and other disease biomarkers [4], as well as environmental pollutants [5], including pesticides [6]. For example, thiabendazole (TBZ), a systemic fungicide used to prevent a variety of vegetable and fruit diseases such as mold, rot, and spots caused by various fungi or to prevent fruit damage [7], was detected using the SERS technique by several authors [8–10]. Despite its detection using conventional methods, such as high-performance liquid chromatography [11], capillary electrophoresis [12] or ion exchange chromatography [13], the limitations of these methods, such as the expensive equipment and need for highly trained operating personnel, time-consuming detection at low concentrations and use of non-eco-friendly reagents and organic solvents, restrict their utilization [14] and require the implementation of faster, efficient, and easier-to-use methods.

In order to achieve reproducible trace analysis, the selection, fabrication, and exploitation of SERS substrates represent a critical and challenging step in developing SERS applications. Stable, uniform, and reproducible substrates that can provide a high Raman enhancement, preferably fabricated by simple, inexpensive methods are highly needed. Metal colloids in suspension are the most common SERS substrates, especially because they are easy to produce and cost-efficient. Several disadvantages of metallic colloids include instability in time, lack of SERS signal reproducibility, as well as, in some cases, the need for stabilizing agents to prevent aggregation, which, however, may lead to a possible hindrance in the detection of molecules of interest due to SERS contributions from the stabilizing agent. On the other hand, aggregation can lead to the formation of hot spots, where the SERS enhancement factors are higher and it was shown that controlling the number of local field hot spots can optimize the SERS efficiency [15]. An alternative for developing stable hot spots is to deposit nanostructures onto a solid surface using methods such as micropipetting, soaking, self-assembly, or Langmuir–Blodgett [16, 17]. A disadvantage of these substrates is that the generated hot spots are distributed randomly over the surface of the substrate. More advanced fabrication methods, such as electron beam lithography, can lead to highly uniform and reproducible substrates; however, even if the SERS enhancements obtained with such substrates are high, the fabrication process is slow and expensive. Among various techniques of nanofabrication, colloidal-based templating or lithography has become significant since it can produce large-area periodic nanostructures offering moderate-to-high SERS enhancements, while being inexpensive and relatively simple. The technique, introduced first by the Van Duyne group, involves a mask made of self-organized polystyrene nanospheres/microspheres of various diameters and a metal (mainly gold or silver) overcoat [18]. These substrates based on metal film over nanospheres (MFoN) were shown to be useful and reliable for various applications, especially biosensing, offering high SERS signal reproducibility and simple fabrication process. MFoN were employed to detect synthetic dyes in food and drinks [19], molecules of biomedical importance, such as glucose [20], allergens in food [21], as well as environmental pollutants [22]. Various approaches to increase the surface roughness and increase the number of hot spots on MFoN have also been proposed. A recent development that involved dealloying showed that by controlling the pore and gap size between the nanoparticles more than 35 times larger SERS enhancement compared to conventional MFoN substrates can be obtained due to large surface area and abundant hot spots [23]. Another performance-enhancing method was based on microwave synthesis to obtain silver nanoparticles/AuFoN SERS substrates, which allowed trace detection of melamine [24].

For several SERS applications, the nanostructured substrates need to be immersed or in contact with water/ aqueous environment. Among these, two categories have recently attracted a lot of attention: microfluidic SERS and electrochemically assisted SERS (EC-SERS). In microfluidic SERS, integration of SERS substrates with microfluidics allows, first of all, a controlled introduction of the analyzed sample over the SERS sensor surface, which can significantly increase the control over experimental conditions, offering the ability to improve the SERS sensitivity, reproducibility, and limit of detection in sensing. Therefore, microfluidic SERS has the potential to overcome some inherent drawbacks of the SERS technique, by bridging the gap toward integrated SERS lab-on-a-chip devices, and thus becoming a powerful, more widely accepted and versatile tool in analytical chemistry [25]. In EC-SERS experiments, the typical goal is to measure the Raman/SERS spectra of molecules adsorbed chemically or physically at the surface of a nanostructured electrode [26]. In recent years, EC-SERS has been developed into a potent surface analysis technique for the in situ analysis of adsorption behaviors and chemical changes at electrode surface. This technique has been used to understand chemical/physical processes at liquid–solid interfaces or to study the arrangement and orientation of molecules adsorbed on well-defined electrochemical surfaces [27]. Among the more practical EC-SERS applications, the detection of pesticides or other pollutants from environmental waters can be mentioned [28–30]. A crucial step in the development of any SERS-based detection application is the optimization of the enhancement efficiency of the SERS substrate. It is well known in plasmonics that the refractive index of the environment strongly changes the optical/plasmonic response of metal nanostructures [31], and at the same time, that direct correlations exist between plasmon resonances and SERS efficiency [32, 33]. Therefore, for developing detection protocols for analytes in solutions, the efficiency of the SERS substrates should be analyzed and optimized in the same solvent (usually water) environment. Although this conclusion appears obvious, such studies, analyzing the plasmon-induced optical response in water, especially for structures with a complex optical response, and correlating it to the SERS efficiency in the same solvent, are absent in the literature.

In this work, we analyze the dependence of the SERS signal of AuFoN on sphere diameter, in both air and water, and correlate them with their optical response in these media. FDTD simulations are performed to allow us to better understand the optical response, surface plasmon-induced electric field enhancement, and the dependence of the SERS enhancement of AuFoN films on their environment. We then explore the possibility to exploit these AuFoN as working electrodes for EC-SERS analysis of thiabendazole, as well as their integration in a flow-through microfluidic channel. The novelty of this work stems from the correlations presented between the plasmon-induced optical response and the SERS efficiency of the AuFoN in both air and aqueous environments. Despite several previous works presented in the scientific literature [34, 35], very few studies focused on describing how the SERS efficiency of AuFoN varies with the size and environment using both experimental investigations and simulations. Moreover, the applicability of the same SERS substrate using various experimental approaches, such as common SERS, EC-SERS, and microfluidic SERS, represents a strong advantage of the AuFoN structures demonstrated in this work. This study constitutes an important step toward the development of EC-SERS in microfluidics devices for sensing applications.

## Experimental/methods

### AuFoN fabrication

Polystyrene (PS) plates of 1 mm thickness were used as substrates. These were washed with ethanol and isopropanol and then dried in nitrogen flow. The plates were treated by UV-ozone for 20 min to hydrophilize their surface and then fixed on a computer-controlled translation stage. Colloidal nanosphere arrays were obtained on the PS plates by the convective self-assembly (CSA) method. Aqueous suspensions of polystyrene nanospheres with different diameters/concentration were used for the AuFoN fabrication: 300 nm/5% w/v, 460 nm/10% w/v, 600 nm /10% w/v,800 nm /10% w/v(Merck/Sigma-Aldrich), 400 nm/5% w/v, 497 nm/2% w/v (Thermo Fisher), and 719 nm/5% w/v (Microparticles). A given volume of colloidal suspension (~ 10µL) was deposited on the substrate along the edge of a rectangular slide fixed in the immediate vicinity of the substrate and inclined at an angle of ~ 25°. The substrate was then translated at the appropriate speed for obtaining monolayers as follows: 30–40 µm/s for the 300 nm spheres, 20–30 µm/s for the 400 nm and 497 nm spheres, 45–60 μm/s for the 460 nm spheres, 40–50 μm/s for the 600 nm spheres, 20–30 μm/s for the 719 nm spheres, respectively, 30–40 μm/s for the 800 nm spheres (see Table S1 in the Supplementary Material for an overview of the fabrication conditions). All films were prepared at ambient temperature and humidity. On top of the PS nanosphere arrays, 120-nm-thick gold films were deposited by magnetron sputtering (Q150R PLUS sputter coater, Quorum Technologies) from a disk-shaped Au target. A 20 mA direct current sputtering process was carried out in a vacuum chamber at a base pressure of 10^−2^ mbar using argon gas, reaching a deposition rate of ~ 2.5 nm/min. The substrate to source distance was about 27 mm with the substrate maintained at room temperature while rotating at a rate of 10 rpm. PS nanosphere arrays of 460 nm were also used for the fabrication of SERS electrodes for EC-SERS measurements. For this, we used a stencil approach: adhesive foils with precut electrode shape were fixed over the nanosphere array. These masks were removed after metal film deposition, leaving behind AuFoN surfaces shaped as electrodes. The nanostructured surface representing the working electrode was manually delimited with nail polish to isolate the immediate gold contact zone.

### AuFoN characterization

Scanning electron microscopy (SEM) images were obtained using a Hitachi SU8230 system operating at accelerating voltage up to 30 kV and magnifications up to 150000×. Size histograms and average size of surface features on the films’ surface were determined by analyzing over 300 nanoparticles in × 100,000 magnification SEM images of each sample by ImageJ open-source image processing software. Optical images and reflectance spectra were recorded through the microscope of a Witec Alpha 300R system. The sample was illuminated through a low numerical aperture (NA = 0.25) objective, and the reflectance spectrum was collected through the same objective. This ensures that the desired area of the sample can be selected and analyzed, while keeping the illumination close to normal incidence. Light was collected by an optical fiber and directed to an Ocean Optics USB4000 spectrophotometer. Measurements were performed in both air and water. For analyzing the reflectance in water, the samples were placed on the bottom of a water-filled Petri dish. The 100% reflectance level is based on the reflectance of an Ocean Optics STAN-SSH high-reflectivity specular reflectance standard.

### SERS measurements

To analyze the SERS efficiency of the different AuFoN samples, p-aminothiophenol (p-ATP) was used as a known Raman reporter, which is covalently bound to gold surfaces. To obtain uniformly coated surfaces, the samples were immersed for 24 h in 10^–4^ M p-ATP in ethanol solution followed by rinsing the samples several times in ethanol. SERS measurements on the AuFoN substrates made of different-sized PS spheres were performed on a Witec Alpha 300R system, using 633 nm and 785 nm excitation lasers and an objective with NA = 0.4. Laser power and integration times/spectrum were 0.25 mW and 10 s (average of 6 accumulations) for the 633 nm laser, respectively, and 2.62 mW and 6 s (average of 6 accumulations) for the 785 nm laser.

### FDTD simulations

The optical/electromagnetic response of the AuFoN was simulated using the Ansys Lumerical FDTD software. A realistic structure, closely mimicking the real samples, was designed, based on a monolayer of polystyrene spheres arranged in a close-packed hexagonal structure on a dielectric substrate. A finite array comprising 90 spheres was considered. The sphere lattice was covered with a 100-nm-thick gold film. The gold triangular nanoparticles formed on the substrate were also included. Material properties were those from Johnson and Christy (1972) for Au (gold). Refractive indices of 1.590, 1.000, and 1.333 were used for polystyrene, air, and water, respectively. Perfectly matched layer (PML) boundary conditions were used for all boundaries. Through the AuFoN and the surrounding region (50 nm above and below), the mesh was defined with a spatial resolution equal to D/100, D being the sphere diameter. Simulations were performed for both air and water media, with D ranging from 300 to 800 nm in 100 nm steps. A plane wave source linearly polarized along the X direction was used to inject light along the Z direction from the sample side. Reflectance spectra were evaluated by placing a power monitor behind the source, while electric fields were mapped by field-profile monitors positioned perpendicular to the AuFoN, in the XZ plane. More information on the fine details of the constructed structure can be found in a recent paper [36], while an image of the 3D model of the AuFoN simulated structure can be found in the Supplementary Material, Fig. S1.

### EC-SERS measurements

In order to build the three-electrode system required for EC-SERS analysis, two external electrodes were used: Ag/AgCl leakless electrode as reference and Pt/Ti wire as auxiliary electrode. A volume of ~ 150 µl of 0.1 mM thiabendazole solution in 0.05 M PBS/0.1 M KCl, pH 7, was dropped on the surface of the working electrode, and the other two electrodes were fixed in a support, making sure that all three are in contact with the solution. The three-electrode cell was placed under a micro-Raman system (AvaSpec-Hero Avantes B.V. Apeldoorn, Netherlands, fitted to a Nikon Eclipse Ci-L microscope) equipped with 785 nm excitation laser. All electrodes were connected at the same time to a portable Sensit Smart potentiostat (Palmsens, Houten, Netherlands). Spectra were recorded under a 20 X objective with a 5.18 mW laser power and 30 s integration times. The surface of the electrode was polarized from 0 to − 1 V (vs. Ag/AgCl) in increments of 0.1 V during SERS measurements.

### Microfluidic SERS

**Fig. 1:**
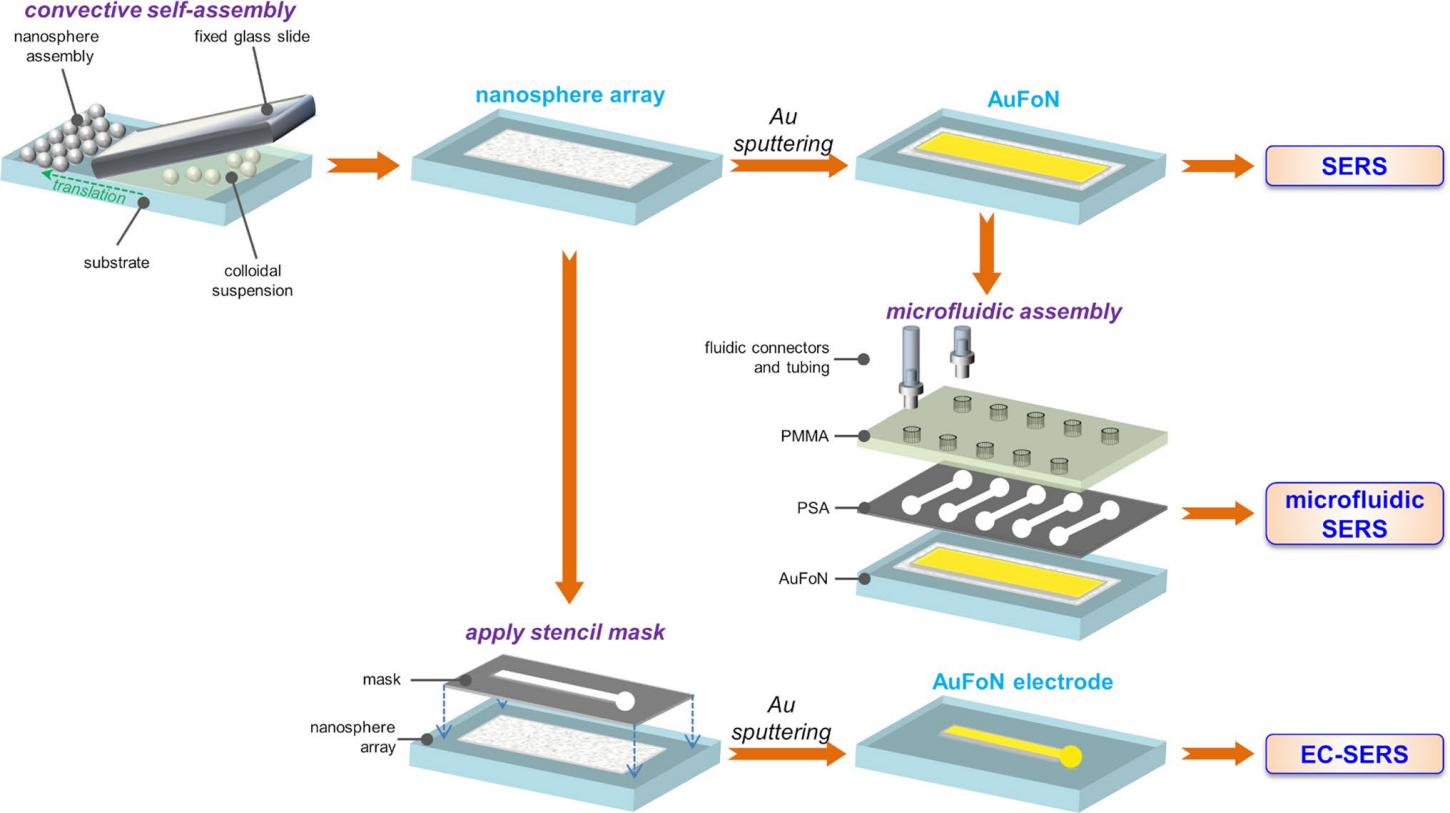
Schematic representation of the process steps for fabricating the substrates for standard SERS, microfluidic SERS, respectively, EC-SERS measurements: PS sphere arrays are obtained by CSA; these are then coated with Au by sputtering to obtain AuFoN and used as such for SERS measurements or embedded in microfluidics; alternatively, AuFoN electrodes are obtained by sputtering through stencil masks and used for EC-SERS experiments

The microfluidic cartridges used in this study were assembled using a bottom layer of PS with immobilized AuFoN, an intermediate layer of pressure sensitive adhesive (PSA: Adhesives Research, Limerick, Ireland) and a 1.2 mm thick top layer of optical quality poly (methyl methacrylate) (PMMA, STRATEC Consumables, Austria) as shown in Fig. 1. 2D design was prepared in AutoCAD 2022. 1 mm wide micro-channels were created in PSA (86 µm thick) using a knife-cutter; inlet and outlet ports in PMMA were prepared using a micro-milling technique. The length of the channels was selected to fit the Raman probe in between, ca. 12 mm. The layers were manually aligned and subsequently laminated by pressing to ensure leak-tight sealing starting from the bottom PS layer.

A programmable Harvard PHD 2000 syringe pump was used for flow control. The 20 mL BD Plastipak TM syringes with luer tips were equipped with a luer adapter and silicon tubing. On the chip’s side, the fluidic connectors (art. 10000700 from Microfluidic ChipShop) were installed in inlet/outlet ports and secured using an adhesive ring (art. 10000717). The flow rate was set at 15 μL/min. The flow was stabilized prior to the measurements for several minutes. For the SERS measurements, i-Raman Plus spectrometer (BWTEK, US) with a fiber probe and 785 nm excitation wavelength was used. The microfluidic SERS device was placed ~ 5.5 mm from the Raman probe (Fig. 1). First, the alignment for SERS was performed in DI water in static flow conditions. Then, TBZ sample in concentration 10^–5^ mM was introduced to the channel and the Raman spectra were recorded using 17 mW laser power and 10 s acquisition time for 30 min, in constant flow. The detection protocol did not require any pretreatment of substrate and sample.

## Results

### AuFoN design by FDTD simulations

**Fig. 2:**
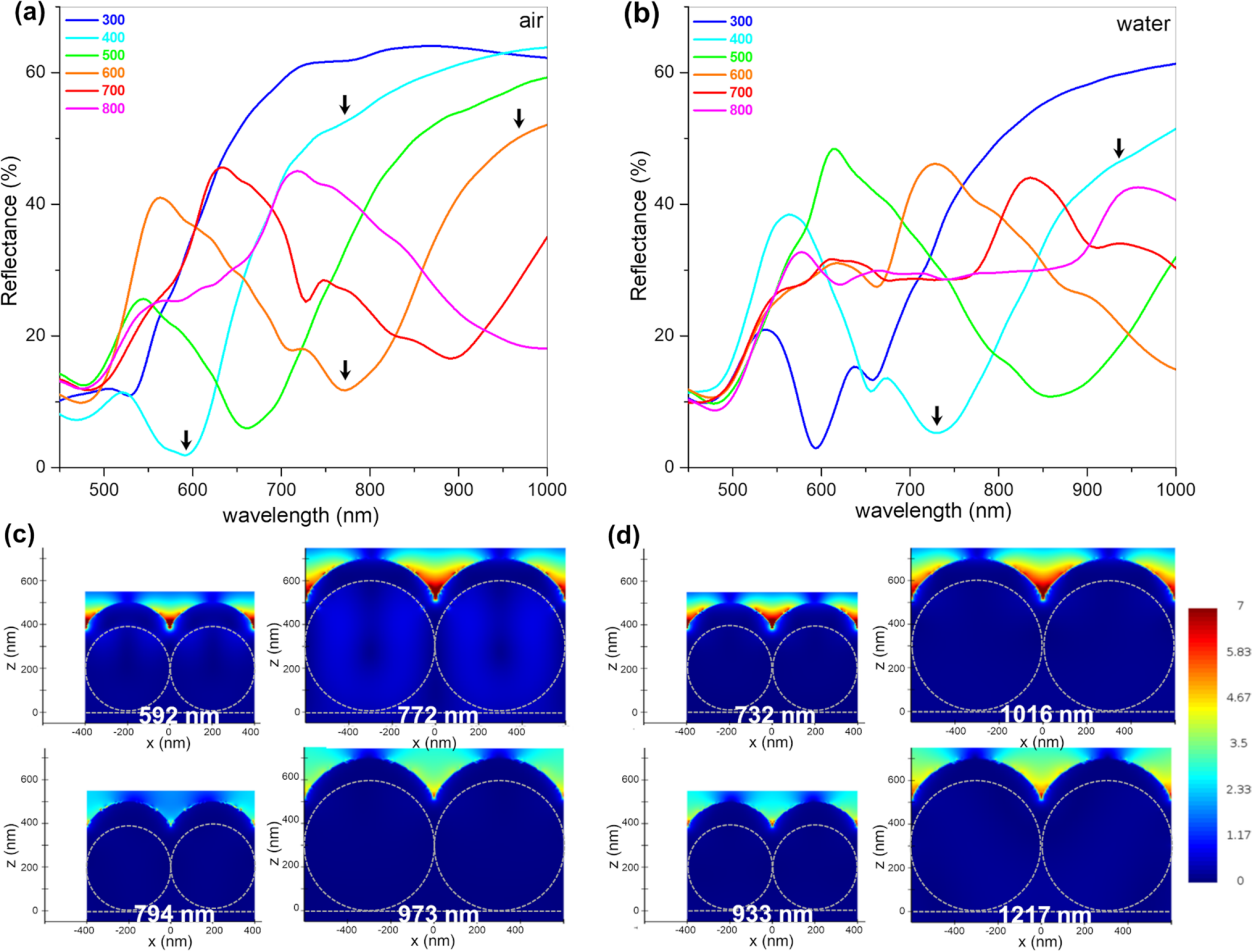
Simulated R spectra for AuFoN made of different-sized (300–800 nm) PS spheres in **a** air and **b** water; Electric field maps in **c** air and **d** water for the AuFoN made with 400 nm (upper) and 600 nm (lower) PS spheres, at the indicated wavelengths corresponding to resonance and off-resonance (arrows in panels **a** and **b**)

The simulated reflectance (R) spectra of AuFoN based on different-sized PS spheres in air and water environments are presented in Fig. 2. The absorption due to the surface plasmons in Au NPs was observed as a broad minimum that red shifts with increasing the diameter of the PS spheres. It is worth mentioning that the transmittance of these structures is negligible for the thickness of the gold film used here; therefore, reflectance is sufficient to be analyzed. For the AuFoN in air, the smallest particles employed in this study (300 nm) showed the plasmonic band (R minimum) at approximately 528 nm, which red-shifted with increasing the diameter until approximately 995 nm for the largest particles of 800 nm diameters. The IR side of the spectrum for the AuFoN800 sample is displayed in Fig. S2 of the Supplementary Material. Additionally, a broadening of the LSPR band was observed with increasing the nanosphere diameter. When AuFoN were immersed in water, the plasmonic band appeared at longer wavelengths compared to the films in air, which can be attributed to the dependence of surface plasmon resonances on the refractive index of their environment. For example, the smallest diameter particles showed a minimum at 593 nm, red-shifted with 65 nm from the AuFoN in air. Larger red shifts were observed for larger diameters of the PS spheres. The 400-nm-diameter particles presented the reflectance minimum at 732 nm, while the 500 nm ones showed a minimum at 857 nm, almost 200 nm red-shifted from the same film in air. Apart from the red-shift shown by the films immersed in water, the shape of the reflectance spectra was similar to the one of the films in air. Next, the electric field distribution on the AuFoN surfaces was analyzed. Electric field magnitudes are presented in Fig. 2c, d for two different PS spheres sizes, 400 nm and 600 nm AuFoN, as well as for two wavelengths (indicated by arrows in Fig. 2a, b): on-resonance, at the reflectance minimum and off-resonance, 200 nm away from the minimum. The electric field presented the highest magnitude on-resonance, while off-resonance, the electromagnetic field decreased considerably. This behavior was present in all AuFoN structures, irrespective of PS sphere size. It can also be observed that the electromagnetic fields generated off-resonance are more intense for the AuFoN made with larger diameter spheres. Additionally, slightly larger electric fields were obtained overall for AuFoN immersed in water compared to those in air. The FDTD results discussed here showed that the reflectance minimum of AuFoN can be easily tuned by the PS sphere diameter, which in turn can be used to maximize the electric fields and thus the SERS efficiency for the desired laser excitation wavelength. More important, the behavior observed in air was also valid in water.

### AuFoN morphology and optical response

**Fig. 3:**
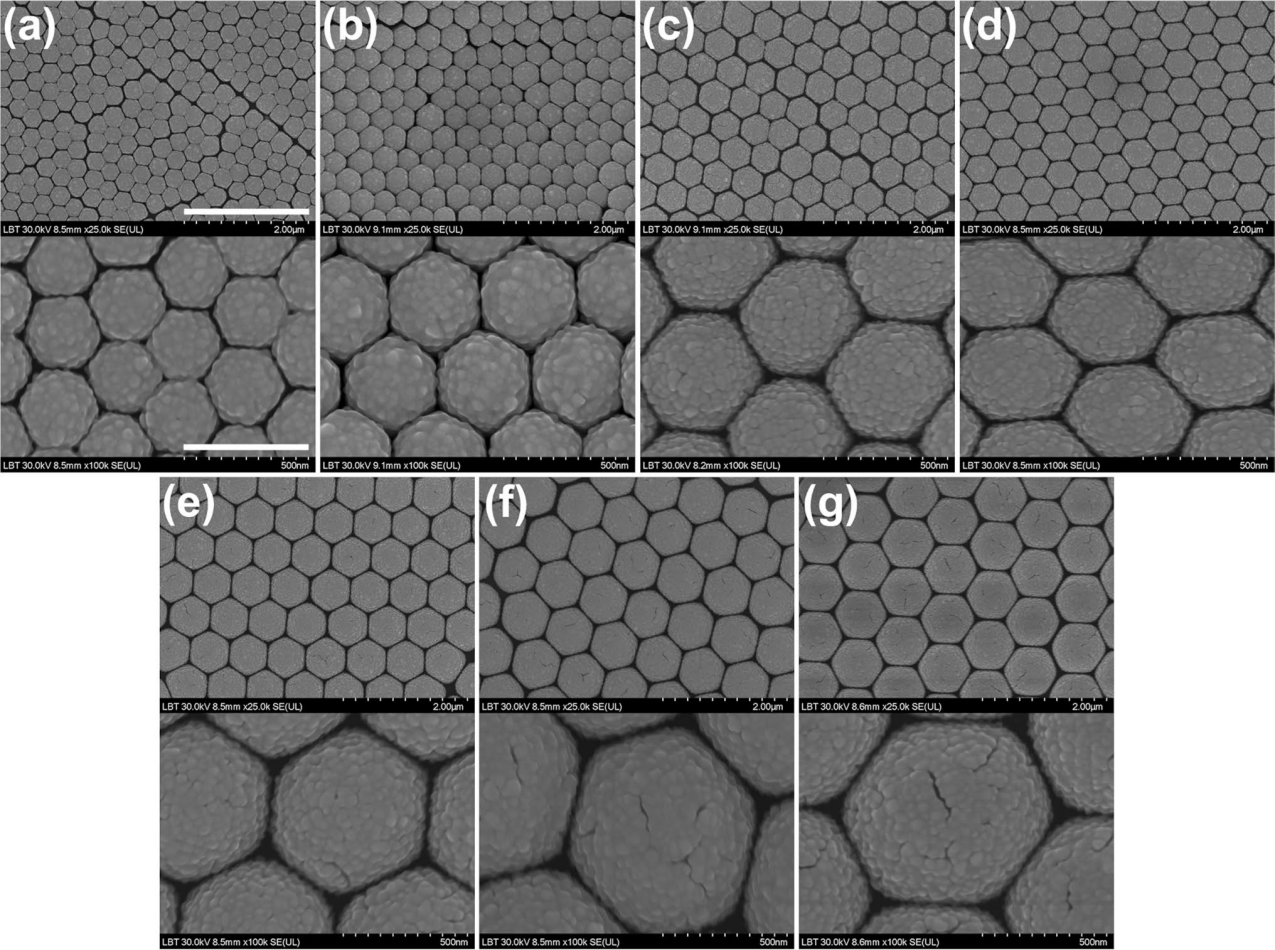
SEM images of AuFoN based on different-sized PS nanospheres: **a** 300 nm, **b** 400 nm, **c** 460 nm, **d** 500 nm, **e** 600 nm, **f** 719 nm, **g** 800 nm. The scale bars inserted on the illustrations in **a** correspond to all images: 2 µm for the ones on top and 500 nm for the bottom ones

In our approach of the CSA technique, the substrate was moved at the desired speed relative to the fixed blade, which also involved the translation of the meniscus (triple air-substrate-suspension contact line) along the substrate. A nanosphere film was thus deposited on the substrate, due to the evaporation of water and the flow of particles from the suspension toward the meniscus. The rate of the colloidal film growth was controlled by substrate translation speed. The thickness of the colloidal crystal film and the number of layers were controlled by adjusting the translation speed and the concentration of particles in the suspension. Figure 3 presents a collection of SEM images on the AuFoN fabricated based on different-sized PS nanospheres. The two-dimensional hexagonal pattern and the gold film roughness inherent to the deposition method and system are visible. The roughness observed on the gold films is determined by the sputtering method and process parameters used on our sputter-coating system. The sizes of the observed particulate surface features were estimated based on SEM images, and size histograms were constructed (Fig. S3 in Supplementary Material). The analysis indicates that the roughness of the film on top of the different-sized AuFoN is similar, in the range 33–40 nm. It is important to note that certain types of defects are inherent to such self-assembled plasmonic substrates. Across the cm^2^ sample area, some regions free of spheres can be found, as well as some regions where patches of double- or multi-layers of the colloidal crystal were formed. In order to avoid such areas, we performed both optical reflectance measurements and SERS measurements through the microscope. Therefore, we can ensure that data and analyses are pertinent to an actual AuFoN formed on a monolayer colloidal crystal.

**Fig. 4:**
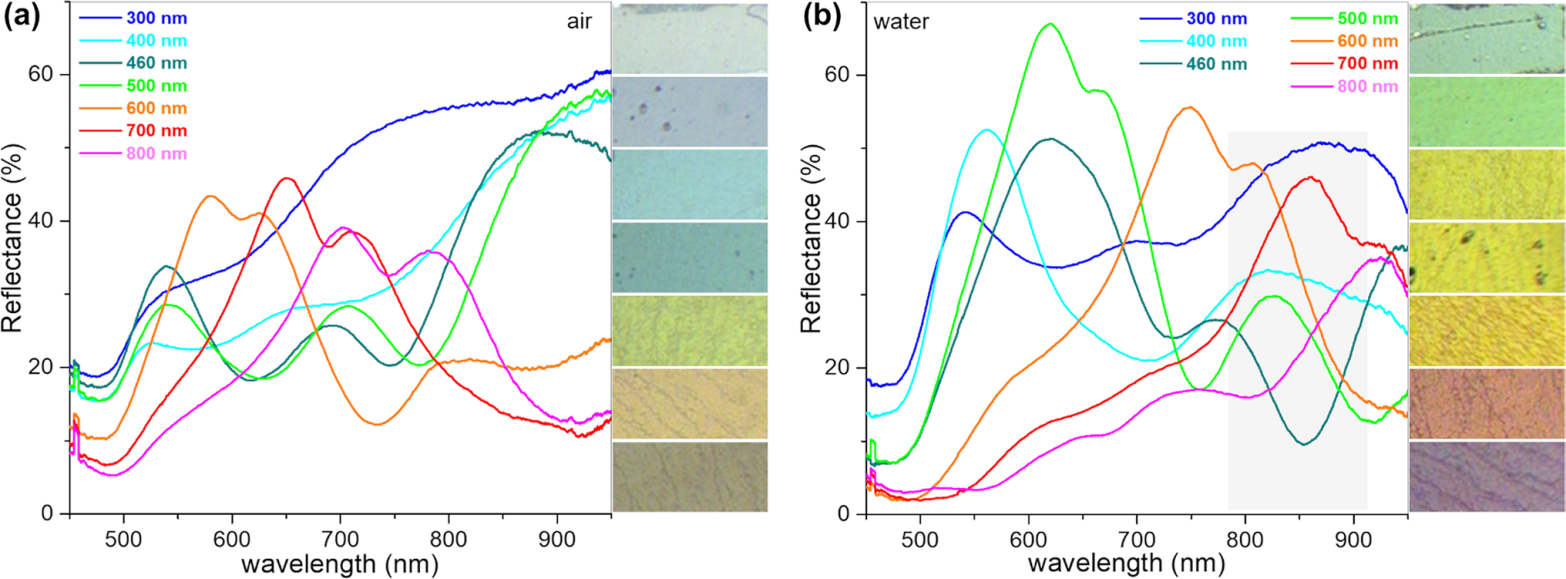
Reflectance spectra for different-sized AuFoN in **a** air and **b** water. On the right side of each figure are optical microscopy images of each sample (images are 200–90 µm)

The reflectance spectra of different-sized AuFoN were measured for the samples in air and water and are presented in Fig. 4. The spectra showed similar behavior to the simulated ones, presenting a reflectance minimum associated with the surface plasmons absorption and increased reflectance toward higher wavelengths. However, the measured spectra are broader, showing two reflectance minima. Both bands present similar dependence on the nanospheres diameters, red-shifting with increasing the size. The dependence of position and intensity of the surface plasmon absorption band on the size of the nanospheres diameter indicated that the position of the band can be tuned by controlling the size of the PS nanospheres used for developing the AuFoN substrates. The intense local electromagnetic fields giving rise to the high SERS enhancement relies on the position of the surface plasmon band; therefore, the choice of the excitation laser line is related to it. In Fig. 4b the region of interest for SERS measurements using a 785 nm excitation line, ranging from 785 nm to approximately 910 nm, is indicated. The positions of minima in the reflectance spectra relative to this region suggests the suitability of the AuFoN films for the 785 nm excitation laser. As demonstrated by other studies, optimal far-field enhancement was achieved when the LSPR was in the spectral range between the excitation line and the Raman bands of interest [37]. As a reference, for the detection of the 1080 cm^−1^ band of p-ATP molecules adsorbed on the surface of the AuFoN immersed in water, the optimal LSPR position would be around 857 nm. Additionally, the reflectance of a flat Au film deposited in the same conditions on polystyrene plates was analyzed. Results indicate a shallow and broad plasmon resonance in the range 600–690 nm (Supplementary Material, Fig. S4). Therefore, we can expect that in this spectral range there is a contribution to the overall response of the AuFoN due to the particulate nature of the film surface, also observed in the SEM images. However, this contribution is considerably weaker than the optical response induced by the geometry and size of the AuFoN.

In Fig. 4, optical images are also displayed, corresponding to the areas used for measuring the reflectance spectra, as observed through the optical microscope. Working under an optical microscope is extremely desirable for these types of studies, since it ensures that both reflectance and SERS spectra are recorded from the same relevant sample areas. It is also interesting to note that the various colors can be associated to the different PS spheres diameter. In air, the smallest nanospheres showed a whitish hue which changed to green, respectively red for the 600 to 800 nm diameter spheres. Similar trend, which can be correlated with the reflectance spectra, was observed for the films in water. The smallest PS nanospheres had a greenish hue, while the largest spheres showed a red to dark-red one. The colors in water are more vibrant due to the better collection efficiency of the setup.

### Tuning the efficiency of SERS in water

In order to provide a complete characterization of the AuFoN SERS substrates, we employed optical microscopy and vibrational spectroscopy. The optical reflectance and SEM investigations revealed the morphology and the dependence of the surface plasmon band on nanospheres’ diameter and environment. Further, to test the enhancement of the fabricated SERS substrates, a small molecule with high affinity to gold was selected. p-ATP is an –SH-terminated molecule, which shows high Au affinity and can bind covalently to the metal nanostructures [38]. Moreover, p-ATP can form a self-assembled monolayer on the metallic surface and due to its characteristic SERS spectrum, the SERS performance of the AuFoN substrates can be analyzed.

**Fig. 5:**
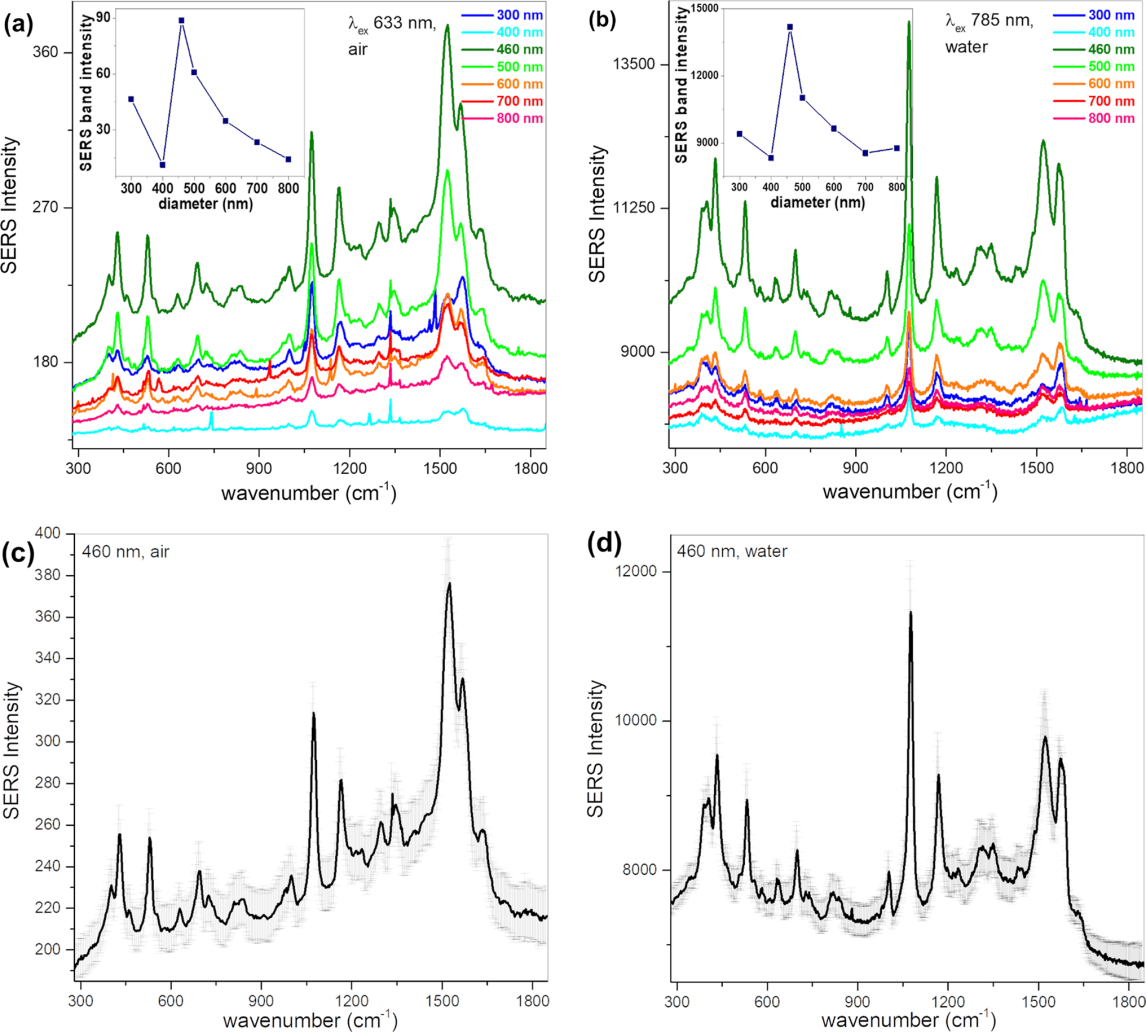
SERS spectra of p-ATP adsorbed on AuFoN of different sizes and excited at **a** 633 nm when samples were in air, respectively at **b** 785 nm when samples were immersed in water. The insets present the dependence of the 1080 cm^−1^ SERS band intensity on the diameter of the PS spheres used for the AuFoN substrates. SERS spectra collected at different locations on the AuFoN460 excited at **c** 633 nm in air, respectively **d** 785 in water

The SERS efficiency of the substrates was analyzed for the films in air and immersed in water, using p-ATP as a Raman reporter. Selected SERS spectra corresponding to p-ATP bound on the different-sized AuFoN are given in Fig. 5. For the water immersion case, the films were excited at 785 nm, as convenient for many practical applications. The inserts in Fig. 5 a and b present the dependence of the intensity of the 1080 cm^−1^ Raman band of p-ATP on the PS nanosphere diameter. As it can be observed, the best signal amplification was given by the 460 nm diameter spheres, which show the closest reflectance minimum to both the 785 nm excitation wavelength and to 858 nm, corresponding to the 1080 cm^−1^ Stokes-shifted Raman band.

Note that the spectral region from 785 to 914 nm (corresponding to the SERS spectrum up to 1800 cm^−1^) is marked by the rectangle in Fig. 4b. The AuFoN500 presents the second-best enhancement, this film exhibiting the reflectance minima closest to the AuFoN460 sample, well overlapping the 785–914 nm range corresponding to the recorded Stokes-shifted Raman spectrum. For the SERS analysis in air, the samples were excited using the 633 nm laser line and the spectra are presented in Fig. 5a. The dependence of the intensity of the SERS band at 1080 cm^−1^ on the nanosphere diameter in air is shown in the inset of Fig. 5a.The AuFoN460 presents the highest amplification, followed by the 500 nm diameter PS spheres. The observed enhancement can be associated again to the location of the reflectance minimum. Both 460-nm- and 500-nm-diameter-sized AuFoN presented the reflectance minima around the 633 nm excitation line and in the spectral region of the recorded Stokes-shifted Raman spectrum. It is important to note that the size dependence in water at 785 nm excitation and the one in air at 633 nm excitation were almost the same. This suggests that measurements in air could be used to calibrate future measurements preformed in water. Obviously, it is more advantageous to perform optimization studies in air, being simpler and more straightforward from the practical standpoint.

Proof of the SERS spectra reproducibility is given in Fig. 5c, d, where several single Raman spectra collected from various locations on the AuFoN460 in both air and immersed in water are shown. Similar band positions and intensities were observed for all spectra. The reproducibility of the SERS bands and their intensity observed in the spectra collected at different positions on the AuFoN suggests that the distribution of p-ATP molecules is similar in different regions of the sample. Moreover, an average enhancement factor (EF) was calculated for the AuFoN460 films excited at 633 nm, by comparing the Raman spectra of p-ATP powder with the SERS spectra of p-ATP adsorbed on the AuFoN. Details for calculating the EF are given in the Supplementary Material. The resulted average EF was on the order of 10^5^, indicating reasonable SERS enhancement even at the 633 nm excitation line. The obtained average EF value can however be considered an underestimate, since only a small sample area actually contributes to the SERS spectra. According to [39], the EF is an average of a broad distribution of enhancement factors resulting from SERS signals obtained from hot spots and “cold” sites, and decreases faster than the power law of local field enhancement, *η*^−1.135^, calculated by La Rue et al*.* for 25 nm diameter Ag spheres separated by 2 nm [40]. Additionally, Fang et al. [39] identified that the cold sites contain more than 60% of the molecules, contributing only 4% to the overall SERS intensity, while hot sites encompass less than 0.1% of the molecules and contribute 24% to the overall SERS intensity. As indicated also by our simulated electric field maps (Fig. 2c, d), the most intense electric fields are generated at the V-shaped grooves/crevices located in between neighboring metallic shells. These field intensities decrease rapidly when moving toward the top of these shells, behavior followed also by the SERS signals collected from molecules adsorbed at these regions. Based on these arguments, a SERS substrate EF [41] in the range 10^6^–10^7^ could be considered more realistic.

### AuFoN as plasmonic electrodes for EC-SERS analyses

**Fig. 6:**
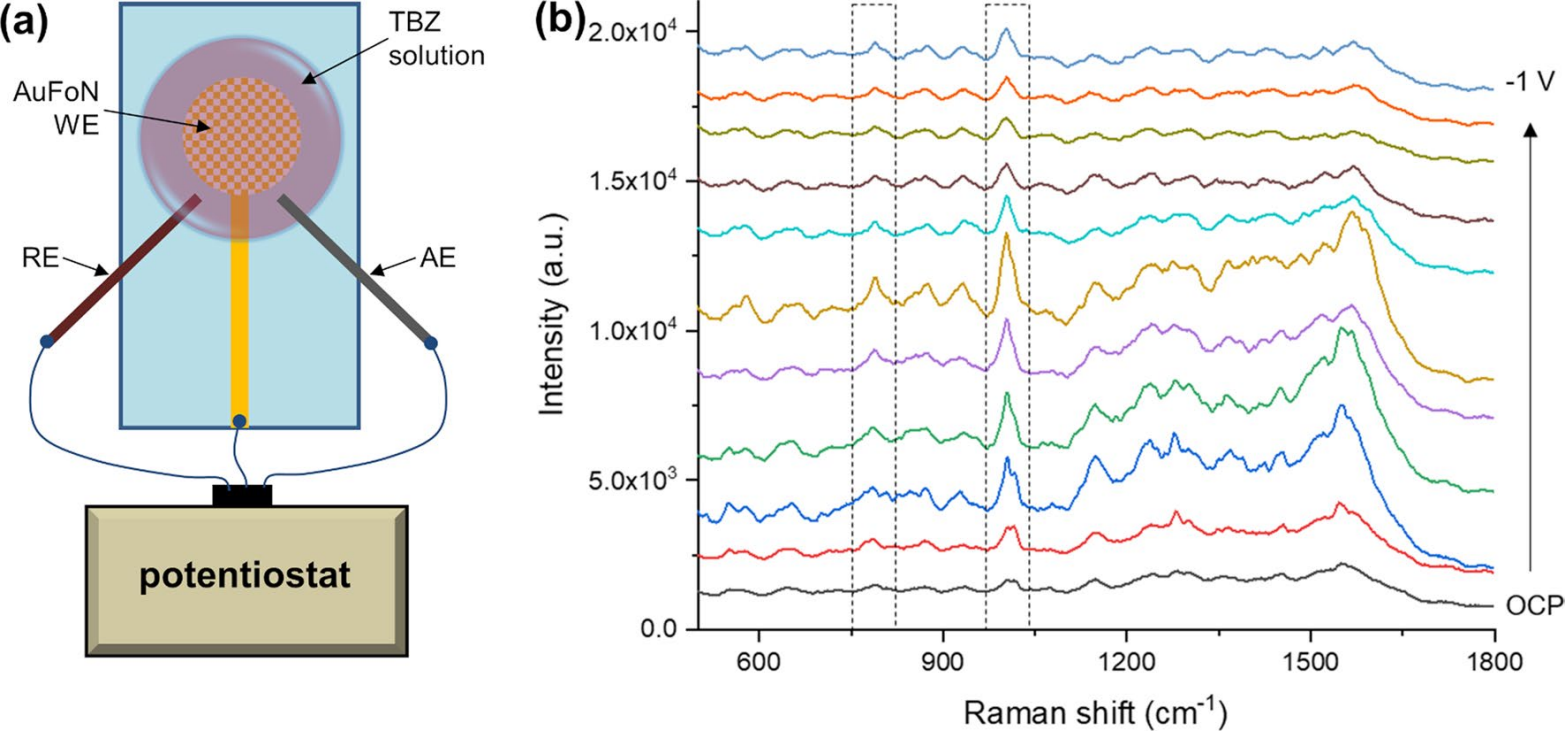
**a** Scheme of the EC-SERS setup configuration. **b** Potential-dependent SERS spectra of TBZ on AuFoN electrode

Next, we explored the possibility to use the AuFoN SERS substrates as working electrodes for EC-SERS, since the combination of these techniques has proven to have various advantages for sensing applications. In EC-assisted SERS, the SERS enhancement is strongly dependent on the applied potential and the molecules present different responses to polarization because they have various electronic configurations. Figure 6 presents the potential-dependent SERS spectra of TBZ recorded on fabricated AuFoN electrodes with external reference and auxiliary electrodes.

The potential-dependent SERS spectra shown in Fig. 6b present Raman bands assigned to both the TBZ molecules, as well as to the substrate. The main Raman bands of TBZ are located at 781, 987, 1012, 1256, 1275, 1303, 1456, 1574, and 1591 cm^−1^, as it can be seen in Fig. S5 of the Supplementary Material. Results indicate that the TBZ Raman peaks were enhanced for the applied potentials in the − 0.3 to − 0.7 V interval, with the highest SERS enhancement obtained at a potential of − 0.5 V (vs. Ag/AgCl). The characteristic Raman vibrational bands of thiabendazole at 787 cm^−1^ and 1002 cm^−1^ [42] presented a distinct increase in intensity. Compared to the SERS spectra obtained at open circuit potential (OCP), the SERS spectra acquired at − 0.5 V, where the highest SERS signal was obtained, the 787 cm^−1^ band was enhanced approximately 1.8 times, while the 1002 cm^−1^ band was enhanced about 3 times. Other TBZ Raman bands detected at the highest SERS enhancement potential were located at 580, 1484, and 1569 cm^−1^. The results obtained from these experiments suggest the means to enhance the sensitivity of TBZ SERS detection by electrochemical effects, which will serve for improving the limit of detection in future practical applications.

### SERS in microfluidics with AuFoN

**Fig. 7:**
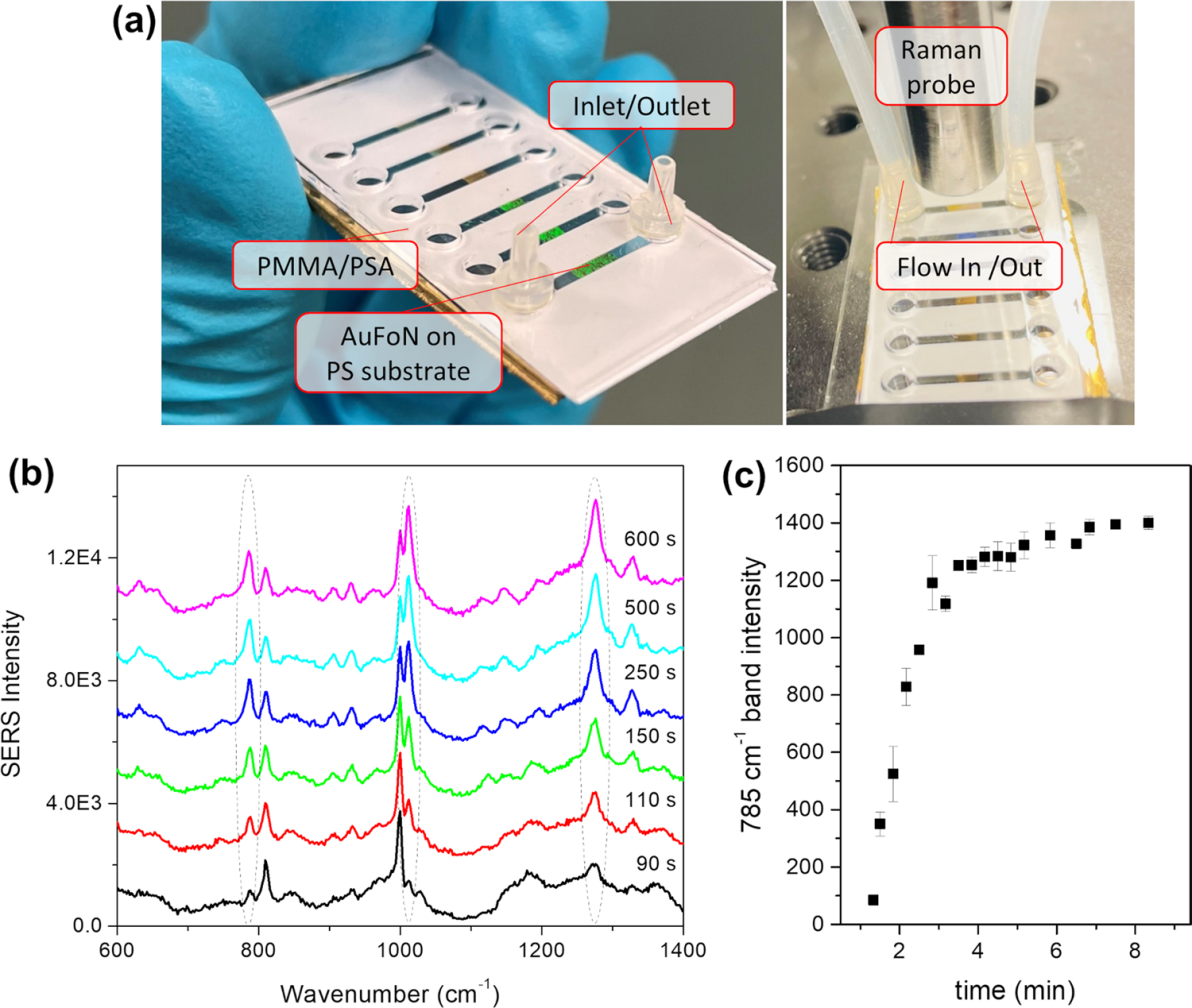
**a** Photograph of the microfluidic SERS assembly (left) and the same assembly under a Raman probe connected to the flow circuit; **b** selection of SERS spectra of TBZ recorded during analyte solution flow; **c** time evolution of the intensity of the 785 cm^−1^ TBZ SERS band

Further, we explored the possibility of integrating the AuFoN SERS surfaces in a flow-through microfluidic channel. The assembly approach we chose is based on the straightforward prototyping, cutting, and laminating of the microfluidic layers, which is a proven strategy for fabricating electrochemical microfluidic devices due to its low costs, versatility, scalability and compatibility with different electrode and substrate materials [43]. Photographs of the realized microfluidic SERS assembly, respectively the assembly under a Raman probe and connected to the flow circuit, are given in Fig. 7a. The AuFoN can be observed as the colored area visible through the PMMA transparent window. Additionally, the inlet and outlet ports in the PMMA layer together with the connected Raman probe are shown. For the microfluidics test, the samples were excited at 785 nm and a TBZ solution was flown through the circuit.

SERS spectra acquired at various time moments are given in Fig. 7b. Characteristic SERS bands of TBZ were detected and increased in intensity with the passing of time and accumulation of the TBZ molecules of interest at the measurement site. These are located at 785, 1012, and 1275 cm^−1^. Additionally, the acquired spectra presented Raman features of the plastic substrate on which AuFoN film was deposited [42]; however, these did not hinder the identification of TBZ pesticide. The intensity of the 785 cm^−1^ band was determined at various time moments and is given in Fig. 7c. It is clear from the time evolution that after a few minutes the intensity of the band saturated. This indicates that either equilibrium has been achieved between the adsorbed molecules of interest and the ones washed by the flow circuit, or the surface of the AuFoN film was fully covered with TBZ molecules.

These preliminary investigations demonstrate that the AuFoN films developed here can be successfully applied for SERS-based applications in aqueous environment. In EC-SERS, the potential-dependent SERS spectra were observed, allowing to further search for the optimal EC conditions, which in turn allow maximizing SERS readout for sensing applications. As for microfluidics detection, the AuFoN films were stable to withstand the flow circuit and the Au nanostructures did not detach with the flow of the liquid, making them attractive for future applications. The results concerning the correspondence between the optical/plasmonic properties in air and those in water can be used during the design and optimization process of a SERS-based sensing device: by monitoring the optical response in air, which is simpler in practice, the response in water could be predicted, and the SERS efficiency can be tuned to the desired spectral range.

In this study, we investigated the SERS performance of the fabricated AuFoN substrates as a function of the PS nanospheres’ diameter, as well as their response in air, respectively, when immersed in aqueous media. The analyses revealed that the highest SERS enhancements were obtained using the 460 nm diameter spheres, which show the closest reflectance minimum to the 785 nm excitation wavelength and the 1080 cm^−1^ Stokes-shifted Raman band of p-ATP, immersed in aqueous media. Further, we showed that microfluidic SERS, as well as EC-SERS detection of TBZ, a common fungicide, can be easily obtained. The aim of this paper was not to test the limit of detection (LoD) for TBZ neither using microfluidic SERS nor with EC-SERS. Our focus was mainly to show that the AuFoN substrates have potential for such applications and that their SERS enhancement is increased under the optimum conditions (immersion in aqueous media, excitation closest to the reflectance minimum, etc.). Further optimization of the experimental configurations for LoD studies is still required and remains to be addressed in future research.

## Conclusions

In this study, we fabricated AuFoN SERS substrates and analyzed their optical response in both air and water environment for PS nanospheres with diameters ranging from 300 to 800 nm. Reflectance spectroscopy measurements revealed the dependence of the LSPR band (i.e., reflectance minimum) on the diameter of the PS nanospheres, red-shifting with increasing the PS sphere size. When immersed in water, the reflectance spectra of these Au nanostructured films retained their shape, while being red-shifted and slightly broadened. FDTD simulations indicated that the strongest electric fields, which are expected to lead to the highest SERS enhancements, in both air and water environments, were located near the reflectance minima. The dependence of the SERS enhancement on the PS nanospheres diameter was evaluated by recording SERS spectra of p-ATP probe molecules, and correlated with the optical properties of the AuFoN. Interestingly, the same dependence was obtained in air and water environment, by changing the excitation laser line from 633 to 785 nm. In each case, the most intense SERS spectra were obtained when the excitation laser line and the spectral region of the Stokes-shifted Raman spectrum were located near the reflectance minimum, as predicted also by simulations. The application of AuFoN as plasmonic electrodes for EC-SERS studies showed promising results enabling the observation of a potential-dependent SERS response of the TBZ pesticide. Furthermore, results confirming the successful integration and use of AuFoN as SERS substrates in microfluidic devices were also presented. The microfluidic-integrated AuFoN enabled the detection of the same TBZ pesticide based on characteristic Raman bands, which showed increased intensity with the passing of time and flow-induced accumulation of TBZ molecules at the measurement spot. Finally, we would like to emphasize once more the importance of analyzing the optical response of nanostructured solid SERS substrates and optimizing it for the desired application in the required environment. Furthermore, our results point toward the perspective of integration of microfluidics and electrochemical SERS analysis in the same miniaturized sensing platforms, which is promising for many applications including medical, life sciences and environmental.

## Supplementary Information

Supplementary file 1

## Data Availability

The datasets generated during and/or analyzed during the current study are available from the corresponding author on reasonable request.

## Code availability

Source code is available upon request from the corresponding author.
