# Supplementary material for: Toward microfluidic SERS and EC-SERS applications via tunable gold films over nanospheres
Source: Discov Nano. 2023 May 3;18(1):73. doi: 10.1186/s11671-023-03851-3 (PMC10214914; doi:10.1186/s11671-023-03851-3)
Supplement: Supplementary file 1 [file 11671_2023_3851_MOESM1_ESM.pdf]

Supplementary Material

Towards microfluidic SERS and EC-SERS applications *via* tunable gold-film-over-nanospheres

Alexandra Falamas[1], Denisa Cuibus[1], Nicoleta Tosa[1], Ioana Brezestean[1], Cristina M. Muntean[1], Karolina Milenko[2], Elizaveta Vereshchagina[2], Rebeca Moldovan[3], Ede Bodoki[3], Cosmin Farcau[1,4*]

[1]National Institute for Research and Development of Isotopic and Molecular Technologies, 67-103 Donat, 400293, Cluj-Napoca, Romania

[2]Department of Smart Sensors and Microsystems, SINTEF Digital, Gaustadalléen 23C, 0373 Oslo, Norway

[3]Analytical Chemistry Department, Faculty of Pharmacy, Iuliu Hațieganu" University of Medicine and Pharmacy, 4 Louis Pasteur, 400349, Cluj-Napoca, Romania

[4] Institute for Interdisciplinary Research in Nano-Bio-Sciences, Babes-Bolyai University, 42 T Laurian, 400271, Cluj-Napoca, Romania

*cfarcau@itim-cj.ro

Table S1. Details of fabrication conditions for the AuFoN substrates concerning the diameter of polystyrene nanospheres, their concentration, and the employed translation speeds for each diameter.

**Diameter of polystyrene nanospheres (nm)**	**Concentration (w/v)**	**Translation speed (µm/s)**
300	5%	30-40
400	5%	20-30
460	10%	45-60
497	10%	20-30
600	10%	40-50
718	5%	20-30
800	10%	30-40

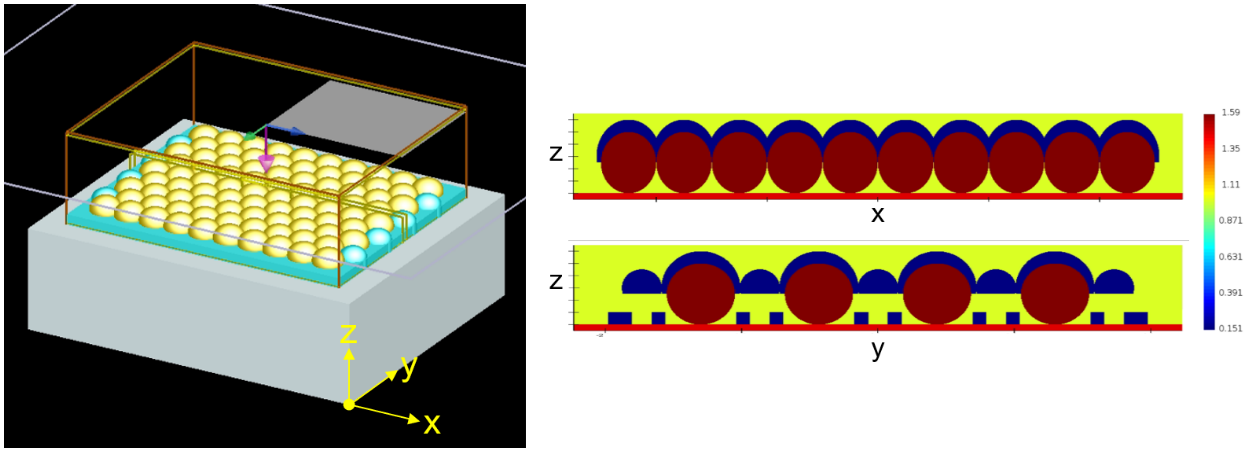

Figure S1. (left) 3D view of the model of the AuFoN employed in the simulations. (right) XZ and YZ cross-sections through the simulated structure.

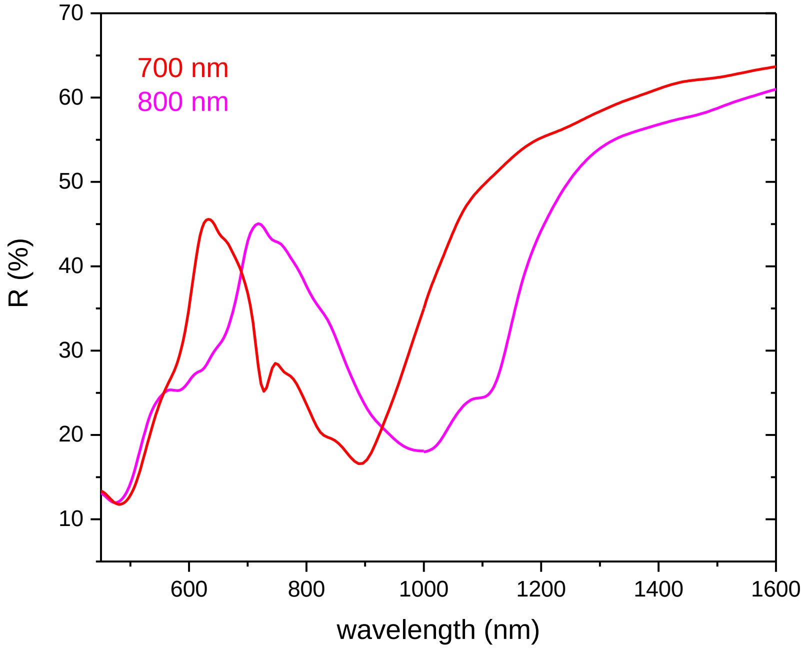

Figure S2. Extended spectral range of the simulated reflectance spectra in Figure 2a of the manuscript (AuFoN made of 700 and 800 nm spheres).

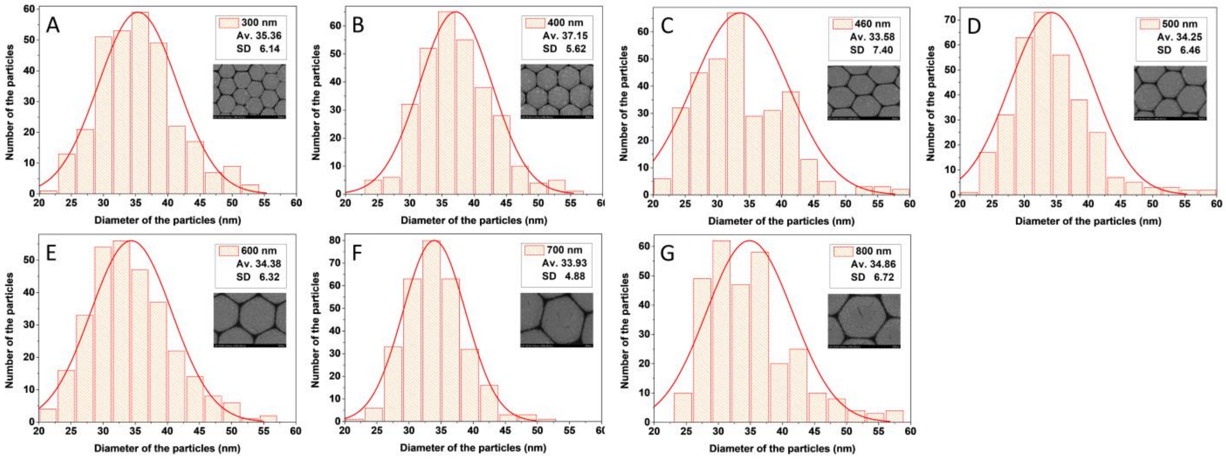

Figure S3. Size distribution of the gold nanoparticles grown at the surface of the gold films deposited on top of 300-800 nm diameter sizes polystyrene spheres (A-G).

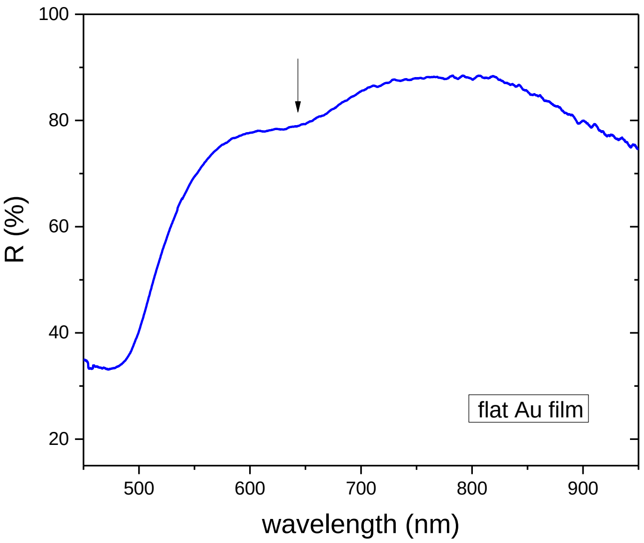

Figure S4. Reflectance spectrum of a flat Au film. The minimum due to roughness is indicated.

SERS EF calculation

The SERS enhancement factor (EF) was calculated using the following formula [J. Phys. Chem. C 2007, 111:13794-803]:

$$EF = \frac{I_{\mathrm{SERS}}/N_{\mathrm{SERS}}}{I_{\mathrm{Raman}}/N_{\mathrm{Raman}}}.$$

Here, I_{SERS} is the intensity (integrated band area) of the SERS band at 1080 cm^{-1}, N_{SERS} the number of excited molecules on the SERS substrate surface, I_{Raman} the intensity of the same band in the Raman spectrum, and N_{Raman} is

the number of molecules in the volume of the focus. Other values involved in the EF calculation are given in this table, for the 633 nm excitation (λ_{exc}): D_f - diameter of the focus spot, A_f - area of the focus spot, H_f - height of the focus (two times the Rayleigh range in the corresponding medium), V_f - volume of the focus, approximated by a cylinder of diameter D_f and height H_f, M_f - the mass of pATP molecule in the focus. For 633 nm laser line, a 20× objective was used with a numerical aperture (NA) of 0.4.

**λ_{exc} (nm)**	**D_f (µm)**	**A_f (µm)**	**H_f (µm)**	**V_f (µm^3)**	**M_f (g)**	**N_{Raman}**	**N_{SERS}**	**I_{Raman}**	**I_{SERS}**	**EF**
633	1.93	2.93	13.87	40.58	4.79E-11	2.30E+11	1.46E+7	7.16E+2	4.20E+3	9.25E+4

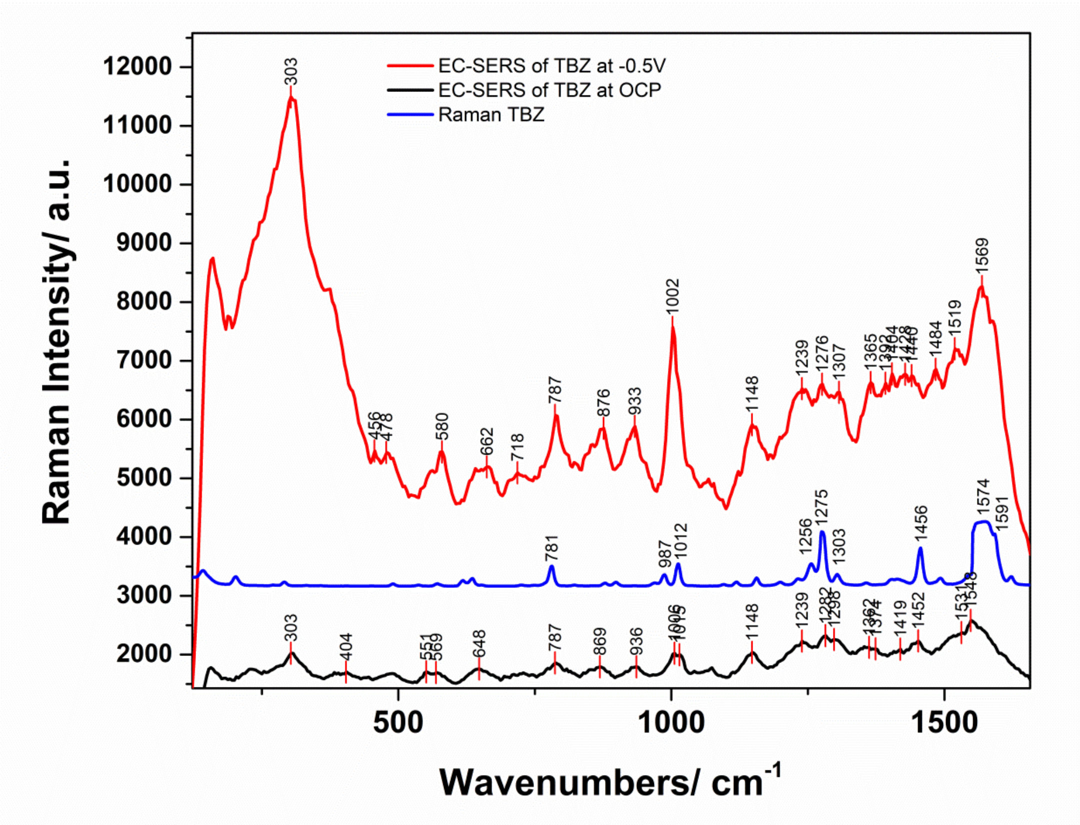

Figure S5. The Raman spectrum of TBZ powder compared to the EC-SERS spectra of TBZ at OCP and at -0.5V, where the highest SERS enhancement was obtained
